# Systemic Lupus Erythematosus and COVID-19

**DOI:** 10.1007/s11926-023-01110-z

**Published:** 2023-07-21

**Authors:** Maria Pappa, Alexandros Panagiotopoulos, Konstantinos Thomas, Antonis Fanouriakis

**Affiliations:** 1 1st Department of Propaedeutic Internal Medicine, Medical School National and Kapodistrian University of Athens, Athens, Greece; 2 4th Department of Internal Medicine, “Attikon” University Hospital, Medical School National and Kapodistrian University of Athens, Athens, Greece; 3 Rheumatology and Clinical Immunology, “Attikon” University Hospital of Athens, Medical School National and Kapodistrian University of Athens, Athens, Greece

**Keywords:** Systemic lupus erythematosus, Covid-19, Vaccination, Outcomes

## Abstract

**Purpose of Review:**

To describe the current state of knowledge regarding COVID-19 in patients with systemic lupus erythematosus (SLE). We focus on (i) SARS-CoV-2 vaccination uptake, immunogenicity and safety, and (ii) outcomes of COVID-19 in patients with SLE and pertinent risk factors for adverse sequelae.

**Recent Findings:**

Notwithstanding the potential concern of patients about possible post-vaccination side-effects, the safety of anti-SARS-CoV-2 vaccines in patients with SLE has been undisputedly confirmed in numerous studies. Humoral immunogenicity is generally attained in SLE, although affected by the use of background immunosuppressive drugs, especially rituximab. The latter has also clearly been implicated with adverse COVID-19 outcomes in SLE, including need for hospitalization, mechanical ventilation and death.

**Summary:**

Although the wide adoption of vaccination has significantly improved COVID-19 outcomes, patients with SLE continue to pose challenges during the pandemic, mainly owing to administered immunosuppressive medications.

**Supplementary Information:**

The online version contains supplementary material available at 10.1007/s11926-023-01110-z.

## Introduction

The coronavirus disease 2019 (COVID-19) pandemic is an ongoing global disease caused by acute respiratory syndrome coronavirus 2 (SARS-CoV-2). The main characteristics of the disease are respiratory distress, fever, pneumonia, cough, fatigue and muscle pain [1–3]. Up until June 2023, the numbers of the COVID-19 pandemic are compelling; more than 767 million people have been infected, and more than 6.9 million deaths have been confirmed [4]. Fortunately, COVID-19-related mortality has decreased substantially during the pandemic, mainly due to increased hybrid immunity from vaccination and prior infections, the circulation of highly infectious but less virulent variants and the advances in the therapeutics of the infection [5]. Nevertheless, the non-linear and unpredictable evolution of SARS-CoV-2, as well as the waning immunity, makes the emergence of new outbreaks possible, even when the virus would become endemic [6].

Current evidence supports that individuals with autoimmune rheumatic diseases (AIRDs) have a slightly increased risk of SARS-CoV-2 infection (relative risk compared to the general population 1.52 (95% CI 1.16–2.00, according to a meta-analysis) [7]. Systemic lupus erythematosus (SLE) is among the rheumatic diseases with the highest risk for infections and related morbidity [8–10]. A variety of immunological dysregulations, including interferon (IFN) pathway, which is also crucial for the innate immune response to SARS-CoV-2 infection, characterize SLE [11]. Interestingly, a recent study using genetic association analysis found shared associated genetic loci between severe COVID-19 and SLE, of which the locus for TYK2 (involved in interferon production) showed the strongest association [12]. Other pivotal studies have shown higher prevalence of mutations in genes involved in type I IFN response, as well as the presence of high titers of neutralizing antibodies against IFN-α2 and IFN-ω in a subset of patients with severe COVID-19 [13, 14]; however, it is not clear if those mechanisms contribute to the increased risk for severe COVID-19 in patients with SLE. In addition to this, patients with SLE often exhibit a more severe net state of immunosuppression due to administered therapies and accumulate higher comorbidity burden, compared to other AIRDs [15].

The rapid development of potent vaccines against SARS-CoV-2 is one of the greatest scientific advances of our time [16], while their safety and efficacy are now well established [17]. However, there is evidence that, after a few months of vaccine availability, the vaccination curve reached a plateau [18]. The hesitancy of patients with AIRDs is mainly due to uncertainty about potential side-effects or flares of the disease [19]. Moreover, the emergence of novel variants is accompanied by significant immune escape from vaccine-induced immunity of novel SARS-CoV-2 variants [20]. As a result, SLE patients, especially those on severe immunosuppression, may still be at risk for infection even after complete vaccination.

In this review, we focus on COVID-19 in relation to SLE, particularly on (i) SARS-CoV-2 vaccination uptake, immunogenicity and safety, and (ii) outcomes of COVID-19 in patients with SLE and pertinent risk factors for adverse sequelae, including the role of administered immunosuppressive therapies at the time of infection, because specific therapies seem to have been particularly associated with adverse COVID-19 outcomes (hospitalization, need for mechanical ventilation, ICU admission and death).

## SARS-CoV-2 Vaccines in Patients with SLE

### Vaccine Acceptance and Uptake in Patients with SLE

Vaccination is the most important preventive measure in our armamentarium against infections, and both the European Alliance of Associations for Rheumatology (EULAR) and the American College of Rheumatology (ACR) regularly update recommendations and guidelines regarding vaccinations in patients with AIRDs [21, 22]. To date, 40 vaccines against COVID-19 have been authorized worldwide by national regulatory authorities, following an unprecedented combined scientific effort by the academia, industry and governmental policies. In a survey including more than 1200 patients with AIRDs (SLE being among the most common, comprising 39% of participants), patients expressed fear of getting severe COVID-19, with a median score of 9 (IQR 7–10) on a scale of 0 (not at all in agreement) to 10 (complete agreement). This notwithstanding, only 54.2% of patients were eager to receive the SARS-CoV-2 vaccine [19], as vaccine hesitancy has been associated with safety concerns about possible adverse events (AEs) and disease flares [23, 24]. In a study evaluating COVID-19 vaccine uptake, unvaccinated patients were more likely to be men (27.3% vs 14.1%), younger (mean 54.1 vs 58.8 years) and to have shorter disease duration (median duration 7.3 vs 10.7 years) [25]. Additionally, they were more likely to have lower rates of influenza and pneumococcal vaccinations, in accordance with evidence suggesting that pneumococcal and influenza immunization rates in patients with AIRDs remain below recommended levels [26, 27]. Thιs observation implies that vaccine hesitancy is not related explicitly to the SARS-CoV-2 vaccine, rather is a generic attitude in a substantial proportion of patients. According to a recent systematic literature review (SLR) and meta-analysis, the pooled vaccine uptake rate in patients with SLE (8 studies, 1168 patients included) was estimated to be 67% (*I*^2^ = 98%), with older patients exhibiting greater acceptance [28].

### Efficacy and Immunogenicity of SARS-CoV-2 Vaccines in Patients with SLE

The efficacy of SARS-CoV-2 vaccines is reflected in the low rates of reported infections following immunization. Of the 5065 SLE patients captured in the SLR of Tan et al. [28], who were vaccinated with at least 1 dose, only 461 (9%) developed COVID-19 infection. It must be noted, however, that in most studies included therein, the follow-up post-vaccination period was relatively short (14–90 days) [29–31], and most were published before the Omicron period of the pandemic. Indeed, with the emergence of the highly transmissible Omicron variants, vaccine efficacy for prevention of symptomatic infection was attenuated, while the protection against severe disease was retained. This was nicely depicted in a recent study from Md Yusof et al. in rituximab (RTX)-treated patients with AIRDs [32•]. The incidence rate before the introduction of vaccines against COVID-19, a period that coincided with the circulation of less infectious variants, was 5.6 per 100 patient-years. After the initiation of vaccination programs, any-severity infections were 89.4 vs 22.8 per 100 patient-years in unvaccinated or partially vaccinated vs fully vaccinated patients; risk reduction was more prominent in the rates of moderate to severe disease (25.5 vs 3.3 per 100 patient-years, respectively).

Regarding cellular and humoral immunogenicity, despite substantial heterogeneity between studies, data are encouraging with pooled seropositivity rates in the Tan et al. SLR reaching more than 80% (81.1%, *I*^2^ = 85%) [28]; of note, mRNA vaccines are associated with higher seroconversion rates compared to non-mRNA vaccines (pooled rates 91.3% for mRNA versus 67.0% and 52.2% for inactivated viral and viral vector vaccines) [28]. Moreover, T-cell response rates seem to correlate with humoral response; however, data specifically in patients with SLE are limited [33, 34].

A major factor that has been associated with reduced seroconversion potential following SARS-CoV-2 vaccination in patients with AIRDs is the underlying immunosuppressive treatment received at the time of vaccination. In this regard, glucocorticoids (GC) [30, 34, 35], mycophenolate [35, 36, 37•, 38•] and combination treatments have been associated with reduced vaccine efficacy in patients with SLE. Moreover, although data specifically for SLE are available in only a small number of patients [35, 36], B-cell depletion therapy with RTX has been consistently implicated with impaired serological response in patients with AIRDs, as these patients show reduced antibody titers and impaired neutralizing activity [35, 39]. Seroconversion is met in less than 50% of patients after 3 doses, whereas even after 4 vaccine doses this rate only reaches 60% [40, 41]. The time elapsed between the last RTX infusion and vaccine administration has been shown to influence antibody titers in some studies, with a longer interval being associated with higher titers [42]. Nevertheless, T cell-mediated immune response is retained in RTX-treated patients, and this may contribute to protection against severe COVID-19, even in the absence of humoral immunity [37•, 43, 44]. Cumulative RTX dose was the main culprit for both humoral and cell-mediated impaired response. Regarding belimumab, data on vaccine immunogenicity are equivocal. Two small studies (in 17 and 30 patients, respectively) found detectable anti-SARS-CoV-2 antibodies in the majority of belimumab-treated patients [45, 46], while in a more recent report from the Hopkins Lupus cohort, belimumab treatment blunted antibody responses in multivariable regression models (a result also found for mycophenolate and tacrolimus). Nevertheless, it should be stated that only 12 of total 334 patients in this study were receiving belimumab at the time of vaccination; thus, results should be interpreted with caution [38•].

An outline of studies related to the effects of background immunosuppression on vaccine efficacy in SLE patients is shown in Table 1. The ACR has provided guidance related to the use and timing of immunomodulatory therapies in relation to COVID-19 vaccination administration in patients with AIRDs [21].

**Table 1:** Administered therapies and response to SARS-CoV2 vaccination in patients with SLE

Author-journal	Design	*N*—vaccine	Therapy received and association
*Naveen, Rheumatology, 2022*	Prospective	532—54% mRNA	No influence of background medication
*Garcia-Cirera, Sci Rep, 2022*	Cross-sectional	39—NS	GC (75% decrease) and RTX (89% decrease) associated with reduced neutralizing antibody levels within 3–6 months (multi)
*So, Ther Adv Musculoskelet Dis, 2022*	Prospective	65 (+ 50 HC)—59%mRNA, 41% inactivated	MMF significantly associated with lower levels of neutralizing antibody (*β* = −15.2, 95% CI −24.4 – −6.0, *P* 0.002); dose of GC significantly associated with lower levels of neutralizing antibody (*β* = −2.01, 95% CI −3.66 – −0.37, *P* = 0.018) (multi)
*Yuki, Arthritis Care Res, 2022*	Prospective	232 (+ 58 non-SLE controls)—Sinovac	GC and MMF use independently associated with lower seroconversion (*P* < 0.001) and NAb positivity (*P* < 0.001) (multi)
*Moyon, Ann Rheum Dis, 2022*	Prospective	126—mRNA	Use of MMF or MTX is associated with reduced vaccine efficacy; use of GC, HCQ, belimumab not associated
*Izmirly, Arthritis Rheumatol, 2022*	Prospective	90 (+ 20 controls)—94.5% mRNA	Use of any immunosuppressant or Pz prior to vaccination-associated with decreased vaccine responses
*Ammitzbøl, ACR Open Rheumatol, 2021*	Prospective	61 (+ 73 RA)—mRNA	Only 4/17 pts (24%) receiving RTX mounted Ab response against SARS-CoV2 (15 RA–2 SLE) (OR 0.07; 95% CI, 0.02–0.26) (multi)
*Boedecker-Lips, Rheumatology, 2022*	Cross-sectional	50 (30 BEL, 20 no BEL)	Only 2/30 pts (6.6%) in BEL group failed to produce Ab against SARS-CoV2 after three vaccinations; they had been previously treated with rituximab. No differences between antibody levels after 3^rd^ vaccination were noted between the two groups (*P*= 0.12) (Wilcoxon exact test)
*Petri, Arthritis Care Res, 2023*	Cross-sectional	342 (12 BEL, 3.5%)	BEL was associated with lower vaccine response (mean SARS-COV-2 spike protein IgG levels) [*β* (95% CI): −2.29 (−4.13, −0.45), *P*= 0.015] (multivariable regression model)
*Fabris, J Autoimmun, 2022*	Cross-sectional	28 (11 RTX, 17 BEL), 13 HC	Median Ab titer was significantly lower in pts receiving B-cell depleting therapy (RTX or BEL) compared to HC (*P*< 0.0001). In 1/11 RTX pt (9%) a low anti-RBD antibodies titre was documented (*P*< 0.0001, compared to HC; however, 8/11 (72.7%) showed IFNγ and IL-2 release comparable to HC (*P*= 0.277). In BEL group, 16/17 (94.1%) pts developed anti-RBD Abs, significantly lower than HC (*P*= 0.002). IFNγ and IL-2 release in BEL group did not differ significantly compared to HC (*P*= 0.036).

### Safety of SARS-CoV-2 Vaccines in Patients with SLE

Notwithstanding the potential concern of patients about possible post-vaccination AEs, the safety of COVID-19 vaccines in patients with AIRDs has been undisputedly confirmed in numerous studies [47, 48•, 49]. In SLE specifically, data are similarly reassuring for mild constitutional flu-like symptoms or localized reactions (injection site pain, rash) usually lasting 7 days, while severe AEs were documented much less frequently [28–30, 33, 50–62]. In the SLR by Tan et al. [28] mild AEs were documented in 44.8% of patients after the 1^st^ dose, and a similar percentage after booster dose [28]; emergency department visit or hospitalization due to major AEs (severe reactogenicity, i.e. high fever, extreme fatigue, severe joint pain) was calculated between 0 and 1.8%, with comparable rates among different vaccine types [31, 61, 62]. No anaphylactic symptoms were reported [61, 62].

Patients’ fear for a disease flare after vaccination is historically the most common reason for vaccination refusal [63]. Nevertheless, the risk of moderate or severe SLE flares (need for hospitalization or change in immunosuppressive treatment) following SARS-CoV-2 vaccination appears to be very low in the literature, between 0 and 2% of patients [28, 29, 34, 50, 56]. Overall, in the aforementioned SLR [28], SLE post-vaccination flares occurred in 5.5% of patients, but the vast majority were mild, mainly referring to mucocutaneous and musculoskeletal symptoms [28, 29, 34, 50, 51, 53–56, 58, 62, 64–67]. Severe flares seem to be extremely rare. Parameters found to correlate with a higher risk for SLE post-vaccination flares are higher disease activity [58], anti-dsDNA positivity [58], experience of flare in the year prior to vaccination [29] and treatment with azathioprine [56] or belimumab [58]. Table 2 summarizes the studies related to SARS-CoV-2 vaccine efficacy and risk for disease flare in SLE patients.

**Table 2:** Summary of studies regarding SARS-CoV-2 vaccine efficacy and risk for disease flare in patients with SLE

Author-journal	Design	*N*—vaccine	COVID-19	Humoral immunogenicity	AE	SLE flares
*Naveen, Rheumatology, 2022*	Prospective	532—54% mRNA	NA	NA	Minor: 83.0%; major: 2.6%, hospitalization: 0.2	NA
*Mok, Vaccine, 2022*	Retro	449 (+465 unvacc.)—61.5% mRNA	NA	NA	NA	8.2% vs 6.2% in unvaccinated, OR 1.40 (0.81–2.43)
*Zavala-Flores, Clin Rheumatol, 2022*	Prospective	100—mRNA	NA	NA	90–92% (severe NR)	9% after 1st, 20% after 2nd dose (2% severe)
*Boedecker-Lips, Rheumatology, 2022*	Cross-sectional	50 (30 BEL, 20 no BEL)	NA	80% after 2, 90% after 3 vaccinations	NA	NA
*Gerosa, Vaccines, 2022*	Retro	452—98% mRNA	17%	NA	26.3%	4% (1% severe)
*Wang, Biomed Pharmacother, 2022*	Cross-sectional	60 (+ 70 RA, 35 HC)—Sinovac	NA	50%	25% (0% severe)	NA
*So, Ther Adv Musculoskelet Dis, 2022*	Prospective	65 (+ 50 HC)—59%mRNA, 41% inactivated	0%	NA	82% (0% severe)	No change in SLEDAI, 0% flares
*Yuki, Arthritis Care Res, 2022*	Prospective	232 (+ 58 non-SLE controls)—Sinovac	4%	62–70%	59% (0% severe)	No change in SLEDAI
*Moyon, Ann Rheum Dis, 2022*	Prospective	126—mRNA	NA	57% (0% severe)	0% severe	2% (0% severe)—no change in SLEDAI or BILAG
*Izmirly, Arthritis Rheumatol, 2022*	Prospective	90 (+ 20 controls)—94.5% mRNA	NA	71%	NA	11% (1% severe)
*Ammitzbøl, ACR Open Rheumatol, 2021*	Prospective	61 (+ 73 RA)—mRNA	NA	89%	NA	NA
*Mormile, Vaccines, 2022*	Prospective	41—mRNA	NA	100%	0% severe	No change in mean SLEDAI (24% increased SLEDAI)
*Yoshida, Lupus Sci Med, 2022*	Prospective	150—mRNA	NA	NA	NA	No increased risk of flares
*Larsen, Clin Exp Rheumatol, 2023*	Prospective	93.5% mRNA	31.7% (all mild)	83% after 2nd dose, 93.5% after 3rd	91% (0% severe)	No change in SLAQ and SDI
*Assawasaksakul, Lupus Sci Med, 2022*	Prospective	71	0%	97%	91% (0% severe)	7% after 4th dose
*Assawasaksakul, Vaccines, 2022*	Prospective	64	NA	81%	86%	0%
*Tang, Clin Exp Med, 2022*	Cross-sectional	188 (+ 190 HC)—Sinovac	NA	NA	44% (0% severe)	1%
*Barbhaiya, Clin Rheumatol, 2022*	Cross-sectional	136—97% mRNA	NA		61–71% (0% severe)	1st dose: 6% (1% severe) 2nd dose: 3% (0% severe)
*Bartels, Clin Rheumatol, 2022*	Cross-sectional	128—mRNA	NA	NA	98%	0% severe flares
*Rider, Rheumatology, 2022*	Cross-sectional	763—74% mRNA			44% (self-reported)	7% self-reported (severity not reported)
*Ferri, J Autoimmun, 2022*	Prospective	38—mRNA	NA	87%	45%	5% (0% severe)
*Fornaro, J Rheumatol, 2022*	Prospective	68—mRNA	NA	NA	53% after 1st dose 67% after 2nd dose Severity not reported	No change in mean SLEDAI
*Felten, Lancet Rheumatol, 2022*	Cross-sectional	696—66% mRNA, 23% inactivated	0%	NA	45% after 1st dose 53% after 2nd dose (17% severe)	3% (2% severe)

A final concern of the public regarding vaccination is the fear of de novo development of an autoimmune condition as a result of aberrant immune activation due to the administered vaccine. In this regard, as with virtually any autoimmune disease, a small number of cases with new-onset SLE after COVID-19 vaccination have been reported to date [68–77]. Importantly, most of them presented with only skin and joint symptoms a few days to 2 weeks after the 1^st^ or 2^nd^ dose of the SARS-CoV-2 vaccine. Most of these patients received GC combined with GC-sparing regimens and demonstrated rapid improvement. In all reported cases, the association between vaccination and subsequent development of SLE (but also any other AIRD) was purely temporal and not etiological. Temporal correlation does not correspond to causative association, and the latter is extremely difficult to establish for rare side-effects of vaccinations [78]. Given the gigantic number of people who have been vaccinated with SARS-CoV-2 vaccines since the latter became available, it is reasonable that several new diagnoses of AIRDs would happen at some point following this vaccination. Along these lines, there has been no report of an increased incidence of SLE after the development of SARS-CoV-2 vaccines, and the very few sporadic cases of new-onset disease should by no means be interpreted as a reason to abstain from vaccination. On the other hand, data from large databases point to an increased risk for new-onset autoimmune or other chronic inflammatory diseases after COVID-19 infection, SLE included [79].

## COVID-19 in Patients with SLE

### Adverse Outcomes in SLE Patients with COVID-19 Infection

#### Hospitalization

Data from the COVID-19 Global Rheumatology Alliance (C19-GRA) registry reported a total of 1922 SLE patients with COVID-19 infection in the period from 03/2020 to 06/2021, of whom the majority (70%) was not hospitalized [80•]. However, when compared to patients with other systemic AIRDs, SLE patients present a higher risk for hospitalization. A Danish observational study showed increased hospitalization risk in SLE patients with COVID-19 compared with the general population after adjustment for age and sex (HR 3.20) [81]. In the pre-vaccination era, Bruera et al. reported a statistically significant increase in hospitalizations in SLE patients compared with general population (31% vs 17.7%) in Houston, USA, but these numbers obviously do not reflect the reality following vaccine availability [82•]. Hispanic ethnicity and black race were associated with increased hospitalization rate compared to white patients (OR 1.73 and 2.15, respectively] [83], while increased risk has been associated with the presence of chronic kidney insufficiency (OR 3.51), cardiovascular disease (OR 1.69) and high disease activity (OR 3.94). Moreover, regarding the potential association between various treatments and hospitalization rates, analyses showed that RTX, MMF and cyclophosphamide (CYC) were associated with increased hospitalization rates (with any ventilation or oxygenation, adjusted OR 1.69, 1.36 and 2.55, respectively) [80]. Even in the high-risk group of RTX-treated rheumatic patients, however, and despite the substantial increase in the total COVID-19 infections during the circulation of Delta and Omicron variants, the incidence of hospitalized cases needing supplemental oxygen in fully vaccinated patients has remained low (3.3 per 100 patient-years) [32•].

Clinicians should be aware of some specific features of COVID-19 in RTX-treated patients, those with SLE included, as they can present with persistent symptoms and long viral shedding or relapsing infection after initial improvement, due to impaired virus clearance by the immune system. There is an increasing number of such cases mostly from patients with hematologic malignancies [84]; however, this syndrome has been also described in SLE patients [85, 86]. What is important regarding these patients, is the within host viral evolution with the acquisition of significant number of mutations in critical genes, such as the one coding for the spike protein, during the protracted course of the infection [87].

#### Mechanical Ventilation, ICU Admission and Death

Need for mechanical ventilation, admission to intensive care unit (ICU) and COVID-19-related deaths seem to be higher in patients with AIRDs than the general population [88•]. Data from C19-GRA reported adverse COVID-19 outcomes in SLE patients to be associated with older age (OR 1.03), male sex (OR 1.5) and comorbidities (OR 1.60)[80•]. In terms of association between specific organ manifestations in SLE and COVID-19 outcome, a small case series of 15 patients with lupus nephritis (LN) reported a mild COVID-19 course in the majority and no severe disease or COVID-associated death, suggesting that LN per se might not increase the risk for COVID-associated morbidity [89]. Finally, two small studies in pediatric SLE populations indicated a benign course or even detection of infection in asymptomatic patients [90, 91]. In general, it should be noted that the substantial reduction in severe COVID-19 and respective mortality seen in the general population [5] reflects also on patients with SLE and other AIRDs [32•].

### Effect of Immunosuppressive Treatments on COVID-19 Outcome in Patients with SLE

Hydroxychloroquine (HCQ), an anchor drug for SLE, was evaluated as an effective treatment for SARS-CoV-2 infection during the early phase of the pandemic [92]. In vitro, HCQ effectively inhibits viral entry, but its use in the clinic was halted due to negative results in randomized clinical trials [93, 94]. In a retrospective cohort study, no difference in COVID-19 infection incidence was noticed between HCQ users and non-users [95]. Similarly, regular HCQ treatment in SLE patients did not result in prevention of COVID-19 infection or amelioration of its manifestations [96–98]. Furthermore, concerns about risk for QT prolongation and increased rate of arrythmias which were noticed in patients with COVID-19 who were treated with antimalarials during the early phase of the pandemic finally resulted in abandonment of HCQ. Mancuso et al. reported a QTc prolongation in 17% of COVID-19 patients treated with HCQ[99], a finding typically not seen in SLE patients chronically treated with the drug [100, 101].

The impact of GC on COVID-19 morbidity in SLE patients attracted considerable attention during the pandemic. An initial report from the C19-GRA found that treatment with a prednisone dose higher than 10mg/day raised the risk for hospitalization by twofold [102]. A more recent report from the same registry showed that even prednisone doses lower than 10mg/day significantly raised the risk for more severe outcomes in multivariable analyses (OR (95% CI) for 1–5mg/day: 1.86 (1.20–2.66); for 6–9mg/day: 2.47 (1.24–4.86); for >10mg/day: 1.95 (1.27–2.99)) [80•]. Additionally, the use of pulse methylprednisolone therapy has been associated with more ICU admissions and increased risk of death [88•, 103, 104]. Contradicting results have also been reported in individual studies; Cortzt et al. found that previous and/or current GC treatment had no detrimental effect in SLE group during the COVID-19 infection, although the authors acknowledged that the number of patients treated with GC was too low in their cohort [81]. Taking all these data into account, the latest EULAR recommendations advise AIRD patients on long-term GC who contract COVID-19 infection to continue GC for reasons of safety and avoidance of a disease flare that could per se contribute to adverse COVID-19 outcomes [105].

Regarding other immunosuppressive treatments, MMF use was not associated with adverse outcomes in multiple different studies [80, 106, 107]. Also, no association has been reported between conventional immunosuppressive drugs (methotrexate and azathioprine) and severe COVID-19-related outcomes [108, 109]. However, data from the RheumaCoV registry from Brazil showed a significant association regarding ICU admission and risk of death with recent CYC therapy (OR 2.26 and 2.86, respectively) [103, 108].

**Fig. 1:**
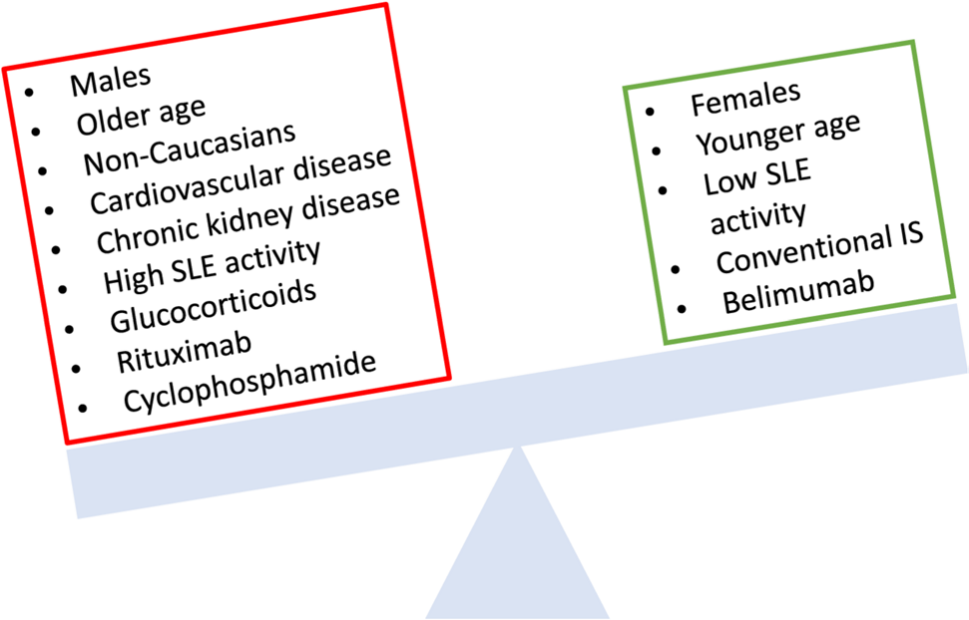
Factors associated with adverse COVID-19 outcomes in patients with SLE. SLE systemic lupus erythematosus, IS immunosuppressive drugs

For belimumab, although a study in Belgium reported higher risk of hospitalization in patents receiving the drug [109], other studies found no independent association [80•]. On the other hand, data on patients treated with RTX are concerning, to say the least. RTX has been clearly associated with higher risk for poorer outcomes in SLE/ COVID-19-infected patients, as reported by the C19-GRA registry. In adjusted models, SLE individuals on RTX experienced worst outcomes regarding hospitalization and ventilation compared to reference group (SLE patients only on antimalarials) (OR 1.69). Similar results were obtained for mechanical ventilation and death [80•, 110–112, 113•]. This increased risk for a more severe COVID-19 course has been confirmed also in various cohorts of patients with other systemic autoimmune diseases treated with RTX, including systemic sclerosis and multiple sclerosis, owing to the blunted humoral immune response conferred by B-cell depleting action [88•, 113•, 114–116], with this risk being higher shortly after RTX infusion. This reality, combined with the fact that, as stated above, patients treated with RTX are also unable to mount protective immune responses following vaccination (even a third booster dose provided marginally improved seroconversion rates in patients with rheumatoid arthritis) [117], renders the decision to treat SLE patients with RTX particularly challenging in the COVID era [118]. Although RTX has provided extremely helpful in certain manifestations of SLE (especially severe, like nephritis, neuropsychiatric disease or arthritis not responding to conventional drugs), patients with lupus have the privilege of alternative immunosuppressive agents, both conventional and biologic, in contrast to their counterparts with e.g. ANCA-vasculitis, wherein RTX is clearly the maintenance therapy of choice. In this regard, we believe it is appropriate to discuss alternative treatment options with SLE patients. Figure 1 depicts the factors associated with adverse Covid-19 outcomes in SLE patients.

The introduction of pre-exposure prophylaxis with the administration of a SARS-CoV-2 neutralizing monoclonal antibodies combination (tixagevimab plus cilgavimab) was an appreciated progress in the prevention of COVID-19 in patients at risk for adverse outcome after infection, or with chronic conditions that preclude a robust immune response to vaccination (a randomized clinical trial showed a 77–82% reduction in symptomatic COVID-19) [119]. Although patients with autoimmune diseases or on immunosuppressive treatments were underrepresented in this trial, real-world evidence supported the efficacy of this strategy in hematologic [120] and rheumatic patients [121]. Unfortunately, FDA deauthorized this product in early 2023, due to significant immune escape of recently emerged SARS-CoV-2 Omicron subvariants and concerns regarding its efficacy in preventing COVID-19 [122]. Depending on the severity of their disease and net state of immunosuppression, SLE patients contracting COVID-19 are candidates to receive antiviral therapy, either nirmatrelvir/ritonavir (as outpatients) or remdesivir (if hospitalized). In such cases, caution is warranted regarding possible interactions between antiviral drugs and background immunosuppressive drugs (Supplementary Tables 1 and 2).

## Conclusions

In conclusion, the COVID-19 pandemic has posed unprecedented challenges for the management of patients with AIRDs, including SLE. Despite the fact that, three and a half years after its appearance, mankind seems to have overcome the initial shock and managed to transform COVID-19 into a controllable public health problem, patients with immunosuppression still represent a distinctive population that merits particular attention. Patients with AIRDs, including SLE, are advised to follow infection prevention and control measures from local authorities, along with regular vaccination against Sars-Cov-2. All things considered, disease severity stratification and net state of immunosuppression depending on administered therapies are important to assess the risk for severe COVID outcomes, among patients with AIRDs, highlighting the need for individualized treatment decisions.

## Supplementary information

ESM 1 (DOCX 17 kb)

## Data Availability

The data from this study will be made available upon reasonable request.
