# Supplementary material for: Systemic Lupus Erythematosus and COVID-19
Source: Curr Rheumatol Rep. 2023 Jul 21;25(10):192–203. doi: 10.1007/s11926-023-01110-z (PMC10504107; doi:10.1007/s11926-023-01110-z)
Supplement: Supplementary file 1 — (DOCX 17 kb) [file 11926_2023_1110_MOESM1_ESM.docx]

**Supplementary data**

**Systemic Lupus Erythematosus and COVID-19**

Maria Pappa^1*^, Alexandros Panagiotopoulos^1*^, Konstantinos Thomas^2^, Antonis Fanouriakis^3^

**Table of Contents**

- Supplementary Table 1: Drug interaction between SLE background immunosuppressive treatment and nirmatrelvir/ ritonavir
- Supplementary Table 2: Drug interaction between SLE background immunosuppressive treatment and remdesivir

| **Supplementary Table 1: Drug interaction between SLE background immunosuppressive treatment and nirmatrelvir/ ritonavir** | | | |
| --- | --- | --- | --- |
|  | **No interaction** | **Potential weak interaction** | **DO NOT**  **co-administer** |
| **Hydroxychloroquine** |  | **X^1^** |  |
| **Methotrexate** | **X** |  |  |
| **Mycophenolate** |  | **X^2^** |  |
| **Azathioprine** | **X** |  |  |
| **Cyclosporine** |  |  | **X** |
| **Tacrolimus** |  |  | **X^3^** |
| **Voclosporin** |  |  | **X^4^** |
| **Rituximab** | **X** |  |  |
| **Cyclophosphamide** |  | **X^5^** |  |
| *^1^ Hydroxychloroquine exposure is increased moderately; not expected to significantly increase the risk of QT interval prolongation.*  *^2^ No dose adjustment is required.*  *^3^ Hold tacrolimus and start nirmatrelvir/ritonavir 12 hours (immediate tacrolimus release) or 24 hours (extended tacrolimus release) after the last tacrolimus dose. Tacrolimus concentrations should be assessed on day 6 or 7 (and every 2-4 days thereafter) and resumption of tacrolimus should begin once drug concentrations approach the therapeutic target.*  *^4^ Related risk of acute and/or chronic nephrotoxicity may increase since voclosporin exposure may significantly increase.*  *^5^ Cyclophosphamide exposure is not anticipated to significantly alter.* | | | |

| **Supplementary Table 2: Drug interaction between SLE background immunosuppressive treatment and remdesivir** | | | |
| --- | --- | --- | --- |
|  | **No interaction** | **Potential weak interaction** | **DO NOT**  **co-administer** |
| **Hydroxychloroquine** |  |  | **X^1^** |
| **Methotrexate** | **X** |  |  |
| **Mycophenolate** | **X** |  |  |
| **Azathioprine** | **X** |  |  |
| **Cyclosporine** | **X** |  |  |
| **Tacrolimus** |  | **X^2^** |  |
| **Voclosporin** |  | **X^2^** |  |
| **Rituximab** | **X** |  |  |
| **Cyclophosphamide** |  | **X^3^** |  |
| *^1^ It may result in reduced antiviral activity of remdesivir. Both drugs have risk of QT prolongation.*  *^2^ Possible risk of QT prolongation.*  *^3^ QT prolongation has been reported.* | | | |
